# Popular science and education of cosmetic surgery in China: Quality and reliability evaluation of Douyin short videos

**DOI:** 10.1111/hex.13737

**Published:** 2023-02-18

**Authors:** Jianfei Zhang, Pengpeng Han, Yujun Tang, Wenwen Xi, Xia Xiao, Feng Yang

**Affiliations:** ^1^ Burn and Plastic Surgery Department, The Second Affiliated Hospital, Hengyang Medical School University of South China Hengyang Hunan China

**Keywords:** cosmetic surgery, Douyin, information quality, medical science, reliability evaluation

## Abstract

**Background:**

Douyin APP is the short video APP with the largest number of users in China.

**Objective:**

This study aimed to evaluate the quality and reliability of short videos about cosmetic surgery on Douyin.

**Methods:**

In August 2022, we retrieved and screened 300 short videos related to cosmetic surgery from Douyin, extracted basic video information, encoded the content and identified the video source. The quality and reliability of short video information were evaluated using the DISCERN instrument.

**Results:**

A total of 168 short videos of cosmetic surgery were included in the survey, and the video sources included personal accounts and institutional accounts. Overall, the total proportion of institutional accounts (47/168, 27.98%) is significantly less than that of personal accounts (121/168, 72.02%); nonhealth professionals received the most praises, comments and even collections and reposts, while for‐profit academic organizations or institutions received the least. The DISCERN scores of 168 short videos of cosmetic surgery were 3.74–4.58 (average 4.22). Content reliability (*p* = .04) and overall short video quality (*p* = .02) are significantly different, but short videos published from different sources have no significant difference in treatment selection (*p* = .052).

**Conclusion:**

The overall information quality and reliability of short videos about cosmetic surgery on Douyin are satisfactory in China.

**Patient or Public Contribution:**

The participants were involved in developing research questions, study design, management and conduct, interpretation of evidence and dissemination.

## 1 INTRODUCTION

In theory, the pandemic of COVID‐19 in recent years will inevitably lead to a decrease in the business volume of the cosmetic surgery industry.^1^ However, on 14 December 2021, the New Oxygen Data Research Institute released the 2021 white paper on medical and beauty industry, showing that 2021 has become the fastest year‐on‐year growth year of cosmetic surgery industry over the past 3 years during the period of repeated epidemics in China. The number of cosmetic surgery customers in China has increased instead of decreasing, and the number of users has increased by about 3 million; one of the driving forces behind this is Douyin APP.^2^ The number of monthly users of Douyin APP exceeds 680 million. It is worth mentioning that these data do not include TikTok, the international version of Douyin. As one of the most successful representatives of China's overseas App, the number of users of TikTok's overseas version is also very large.^3^ With 500 million daily users in China, Douyin has become a breakthrough for cosmetic surgery to attract customers.^4^ The originality, interactivity and sociality of Douyin provide a better user experience and sense of participation for the younger generation while seeking health information.^5^ Douyin offers rich modes of information (such as text, images, audio and video) and contains a large number of technical functions, such as commenting, chatting, tracking, liking and livestreaming. The features make the app easier for the public to use as a source of health information^6^; also, Douyin's penetration and usage among some older age groups are on the rise.^7^ In the years since the advent of Douyin APP, everyone has gradually opened their own horizons, accepted cosmetic surgery more and more and improved their sense of urgency for their own image management.

At present, there are more than 2 million medical institutions engaged in plastic surgery in China, and when we search for ‘plastic surgery’ in Douyin, there are more than 60,000 institutional certification accounts, which means that on average, 3 out of every 100 cosmetic and surgical institutions make short videos in Douyin to attract customers. This is not counting individual users and beauty bloggers. It is conceivable that the competition in the cosmetic surgery industry is fierce, but it also proves that the popular science and education of Douyin short video is an excellent channel for cosmetic surgery institutions to attract customers on the internet.

However, the huge number of users also means that the quality of short videos may be uneven, and the quality and reliability of the information provided by them need to be evaluated. Therefore, the purpose of this study is to evaluate the quality and reliability of Douyin short videos related to cosmetic surgery by investigating the characteristics, sources and contents of related videos in Douyin APP.

## 2 METHODS

### 2.1 Search strategy and data extraction

**Figure 1:**
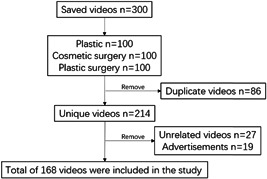
Search strategy and video filtering method.

On 18 August 2022, we searched for the keywords ‘plastic’, ‘cosmetic surgery’ and ‘plastic surgery’ in Douyin APP. Considering that most users use the default value to search results, the ranking basis is ‘comprehensive ranking’, with unlimited release time, unlimited video duration and unlimited search range. According to the search results of each keyword, due to the principle of ‘least effort’,^8^ of the top 100 short videos of keywords are selected, and there are 300 short videos of three keywords. Among them (1) there were 86 repetitions, (2) 27 videos were not related to cosmetic surgery and (3) there were 19 advertisements. A total of 168 short videos were included in the analysis and research after excluding the above videos (Figure 1).

In terms of data collection, in this study, Questionnaire Star (Changsha Ranxing Information Technology Co., Ltd.) was chosen as a professional questionnaire platform to establish an online questionnaire, encode the short video content and classify and count the source, duration, number of likes, comments, collections and shares of short videos (Table 1).

**Table 1:** Videos characteristics (ranking of the top 10 according to the number of short video likes).

Link	Number of likes (*n*)	Number of comments (*n*)	Number of collections (*n*)	Number of forwarded videos (*n*)
https://v.douyin.com/r1rfHyA/	467,537	31,817	13,472	46,951
https://v.douyin.com/r12oEKk/	395,261	34,226	33,218	106,334
https://v.douyin.com/r1hYMMr/	354,675	11,178	35,614	20,053
https://v.douyin.com/r1hjRBr/	271,453	58,662	25,211	97,335
https://v.douyin.com/r1b7Mmw/	247,542	32,337	2591	1184
https://v.douyin.com/r1geTaU/	242,814	8786	4803	16,457
https://v.douyin.com/r1bEmBm/	217,628	12,443	16,715	11,521
https://v.douyin.com/r1bTENX/	202,549	28,667	19,114	43,496
https://v.douyin.com/r1ghqEj/	188,725	3853	1578	577
https://v.douyin.com/r1gketx/	119,475	7110	2448	5666

The DISCERN instrument was used as a method to evaluate the information quality and reliability of each short video. The DISCERN instrument is a statistical scale developed by the Oxford University research team that is used to evaluate the information quality and reliability of the content related to health science education.^9^ The scale consists of 16 questions and 3 sections: assessment of the reliability of the publication content (8 questions); choice and information about treatment (7 questions) and overall quality assessment of publications (1 question). Each question is graded from a maximum score of 5 (absolutely yes) to a minimum score of 1 (absolutely not). Finally, the total score of short videos is 16–80. The higher the score, the higher the quality and reliability of short video information (Table 2).

**Table 2:** The DISCERN questionnaire.

No.	Question
1	Are the aims clear?
2	Does it achieve its aims?
3	Is it relevant?
4	Is it clear what sources of information were used to compile the publication (other than the author or producer)?
5	Is it clear when the information used or reported in the publication was produced?
6	Is it balanced and unbiased?
7	Does it provide details of additional sources of support and information?
8	Does it refer to areas of uncertainty?
9	Does it describe how each treatment works?
10	Does it describe the benefits of each treatment?
11	Does it describe the risks of each treatment?
12	Does it describe what would happen if no treatment is used?
13	Does it describe how the treatment choices affect the overall quality of life?
14	Is it clear that there may be more than one possible treatment choice?
15	Does it provide support for shared decision making?
16	Based on the answers to all of these questions, rate the overall quality of the publication as a source of information about treatment choices

DISCERN scores were independently counted and scored by two senior doctors who have been engaged in plastic surgery for many years. The overall rating agreement (Cohen *κ*) was 0.821 (*p* < .001), which indicated that the rating process had satisfactory interrater reliability. Any between‐group analysis regarding DISCERN scores was performed using the Kruskal–Wallis *H* test in SPSS 22.

## 3 RESULTS

### 3.1 Video characteristics

Video sources include personal accounts (health professionals, nonhealth professionals) and institutional accounts (for‐profit academic organizations or institutions, nonprofit academic organizations or institutions and news organizations). Among them, health professionals accounted for the highest proportion (87/168, 51.79%), followed by nonhealth professionals (34/168, 20.24%). In institutional accounts, the number of for‐profit academic organizations or institutions (29/168, 17.26%) are much larger than nonprofit academic organizations or institutions (5/168, 2.98%) and news organizations (13/168, 7.74%). Overall, the total proportion of institutional accounts (47/168, 27.98%) is significantly less than that of individual users (121/168, 72.02%) (Table 3).

**Table 3:** Characteristics of the sources of cosmetic surgery‐related Douyin videos (*n* = 168).

Source	Description	Videos, *n* (%)
Individual users		121 (72.02)
Health professionals	Medical professionals, including doctors, nurses, technicians, and so forth	87 (51.79)
Nonhealth professionals	People other than medical professionals, including patients and propagandists, and so forth	34 (20.24)
Organizational users		47 (27.98)
For‐profit academic organizations or institutions	Private capital‐operated organizations or hospitals	29 (17.26)
Nonprofit academic organizations or institutions	Organizations or hospitals operated by the state for public welfare	5 (2.98)
News agencies	Organizations provide news coverage	13 (7.74)

Statistical analysis shows that the short video duration of cosmetic surgery ranges from 23 to 450 s, among which news organizations publish the longest short video duration of cosmetic surgery, followed by nonprofit academic organizations or institutions, and health professionals publish the shortest short video duration. The total average time is generally no more than 5 min. As of the time of the study, 168 short videos of cosmetic surgery received 15,682,542 likes, 1,276,879 comments, 1,052,488 collections and 2,685,527 shares. Each short video received from 0 to 467,527 likes, from 0 to 34,226 comments, from 0 to 35,472 collections and from 0 to 106,334 shares. Nonhealth professionals received the most likes, comments, collections and shares, while for‐profit academic organizations or institutions received the least (Table 4).

**Table 4:** Characteristics of cosmetic surgery‐related Douyin videos (*n* = 168).

Source	Duration (s), median (IQR)	Number of likes, median (IQR)	Number of comments, median (IQR)	Number of collections, median (IQR)	Number of forwarded videos, median (IQR)
Individual users					
Health professionals	94 (69–186)	16,542 (2417–31,457)	2757 (651–15,321)	2821 (354–11,024)	4353 (1362–20,767)
Nonhealth professionals	227 (85–297)	85,184 (8754–181,612)	8214 (587–26,417)	6804 (349–8995)	58,786 (3354–85,133)
Organizational users					
For‐profit academic organizations or institutions	264 (71–324)	24,327 (1334–36,725)	324 (57–1037)	288 (39–2917)	157 (78–289)
Nonprofit academic organizations or institutions	176 (54–271)	22,436 (4872–36,414)	891 (152–3721)	757 (319–1870)	862 (223–7544)
News agencies	438 (124–473)	15,432 (9434–43,571)	258 (47–1364)	244 (65–2011)	537 (227–2197)
Overall	253 (152–342)	58,724 (20,175–73,427)	3084 (1131–5703)	3758 (624–6397)	4950 (761–29,593)

Abbreviation: IQR, interquartile range.

### 3.2 Short video information quality

As of the time of study, the DISCERN scores of 168 short videos of cosmetic surgery in the survey ranged from 3.74 to 4.58 (mean 4.22). The reliability score of short video content (eight questions) was 3.32–4.36 (mean 3.87). Scores for information on treatment options (seven questions) ranged from 3.55 to 3.67 (mean 3.62). The average score of the overall quality evaluation (one question) of short videos was 3.95. Among them, the short videos released by health professionals had the highest reliability, and the information provided about the choice of treatment was of the highest quality. However, videos released by nonhealth professionals are less reliable, and short videos released by news organizations rarely mention treatment options. There was no significant difference in treatment choice between short videos released from different sources (*p* = .052), and DISCERN scores of short videos of cosmetic surgery released from different sources showed a significant difference (*p* = .01) (Table 5).

**Table 5:** DISCERN scores of cosmetic surgery‐related Douyin videos (*n* = 168).

Category	Health professionals (*n* = 87)	Nonhealth professionals (*n* = 34)	For‐profit academic organizations or institutions (*n* = 29)	Nonprofit academic organizations or institutions (*n* = 5)	News agencies (*n* = 13)	*p* Value^a^
Publication reliability						.04
Median	34.5	29.5	30	33	32	
Mean (SD)	33.4 (1.23)	31.2 (3.27)	30.7 (1.31)	32.1 (1.42)	31.4 (4.91)	
Treatment choices						.052
Median	27.5	22	25	27	21	
Mean (SD)	25.4 (3.60)	22.1 (3.71)	25.2 (4.50)	26 (4.23)	21.1 (6.22)	
Overall quality						.02
Median	4	4	4	4	4	
Mean (SD)	3.5 (1.20)	4.2 (0.91)	3.7 (0.82)	3.9 (0.90)	3.8 (0.77)	
Total score						.01
Median	66	57	61	67	65	
Mean (SD)	64 (5.6)	56.5 (11.2)	57 (11.7)	66.5 (3.3)	64.5 (8.9)	

^a^ *p* values were calculated using the Kruskal–Wallis *H* test.

## 4 DISCUSSION

### 4.1 Health education information on the internet

With the rise and advancement of the internet, information related to health education is flooding the network.^10^ Increasingly more people, in particular, young people tend to look for information about health education and disease diagnosis and treatment on the internet.^11^ Previous studies have found that most of the information about health education on the internet, such as Baidu Encyclopedia and Wikipedia, is one‐sided and incomplete, which can easily lead to misdiagnosis and negative emotions, and may eventually lead to delayed treatment of diseases.^12^, ^13^, ^14^

However, in recent years, things have been changing for the better. Due to the rapid development of mobile internet in China, increasingly more health professionals participate in health education and disease diagnosis and treatment on the internet, and a large number of professional health science popularization websites and online diagnosis and treatment websites have developed, such as Tencent Medical Code (Tencent Technology Co., Ltd.), Chunyu Doctor (Beijing Chunyu Tianxia Software Co., Ltd.), and so forth, which include topics related to health education as well as professional disease prevention, diagnosis and treatment.^15^

There is evidence that, during the Chinese novel coronavirus pneumonia pandemic, due to the epidemic situation, most people chose to reduce the number of activities away from home. In order to pass the time, there are a huge number of short videos of different types on Douyin, which can meet the interests and viewing needs of different groups of people.^16^ Douyin has become the most used short video APP in China, so Douyin has great communication potential.^17^ A large amount of health education‐related information can be disseminated through Douyin, which makes it more convenient and easy for ordinary people to obtain health education information. However, the huge number of users also means that the quality of short videos may be uneven, and the quality and reliability of the information provided by them need to be evaluated.

### 4.2 Principal findings

A total of 168 short videos of cosmetic surgery were included in the survey, and the video sources included personal accounts and institutional accounts. On the whole, the total proportion of institutional accounts is significantly less than that of personal accounts. In personal accounts, the proportion of health professionals is high, which is mainly due to the rapid development of We Media, which makes more and more professionals aware of the knowledge differences between themselves and the public. Through this difference, they can use short videos as tools to expand their personal influence, and finally bring economic and social benefits to themselves.

Nonhealth professionals received the most praises, comments and even collections and reposts; this is mainly because a large number of internet celebrities and popular stars also need to attract fans and audiences through different short video contents, and cosmetic surgery can arouse the audience's curiosity. At the same time, millions or even tens of millions of fans also bring a huge number of praises, collections, comments and reposts to these accounts, such as the account name ‘Hangjun Wang’. Although he is not a health professional, through a series of short videos on beauty, makeup and skin care, he gained 786,000 fans on the Douyin, so he released a short video about the failure of cosmetic surgery on 31 July 2021; about 395,261 likes, 34,226 comments, 33,218 collections and 106,334 shares were gathered (https://v.douyin.com/r12oEKk/). Although the DISCERN score, which evaluates the quality and reliability of short video content, is not as high as that for the short videos released by health professionals, the communication effect of short videos is difficult for ordinary health professionals to achieve. However, for‐profit academic organizations or institutions receive the least number of praises, collections, comments and reposts, which may be related to the decrease of patients' trust caused by the chaotic cosmetic surgery results of for‐profit organizations.

Overall, the reliability and overall quality of 168 short videos of cosmetic surgery were high, but there was no significant difference in treatment choices between short videos released from different sources.

### 4.3 Limitations and future research

There are some limitations in this study. First of all, we only use the DISCERN instrument; however, there are other tools available, such as the *Journal of the American Medical Association* benchmark criteria,^18^ the Global Quality Score^19^ and the HONcode.^20^ Future research can expand these studies by using different tools, and comprehensive utilization of more evaluation tools can lend more credibility to the results. Second, this study only evaluated the information quality of short videos of cosmetic surgery in China. Although the location does not affect the overall results, the conclusions may not be applicable to short videos in other languages. Therefore, we call for more cross‐language comparative studies in the future.

## 5 CONCLUSION

The overall information quality and reliability of short videos about cosmetic surgery on Douyin are satisfactory in China. However, when watching short videos related to cosmetic surgery on Douyin, patients should choose carefully because different video sources have different emphasis on the introduction of cosmetic surgery. To find accurate health information, it is important to seek the help of medical professionals, rather than looking for information provided on the internet in chaos. Finally, based on the limitations of this study, we put forward some suggestions for future research.

## AUTHOR CONTRIBUTIONS

Feng Yang and Jianfei Zhang designed the research. Jianfei Zhang and Pengpeng Han performed the research. Yujun Tang and Wenwen Xi analyzed the data. Jianfei Zhang and Xia Xiao wrote the paper.

## CONFLICT OF INTEREST STATEMENT

The authors declare no conflict of interest.

## Data Availability

The data that support the findings of this study are available from the corresponding author upon reasonable request.
